# Identification of a Monoclonal Antibody against Porcine Deltacoronavirus Membrane Protein

**DOI:** 10.3390/ijms241813934

**Published:** 2023-09-11

**Authors:** Huiguang Wu, Chen Li, Xian Sun, Yue Cheng, Zhenhai Chen

**Affiliations:** 1 College of Veterinary Medicine, Yangzhou University, Yangzhou 225009, China; 2 Joint International Research Laboratory of Agriculture and Agri-Product Safety, The Ministry of Education of China, Yangzhou University, Yangzhou 225009, China; 3 Jiangsu Co-Innovation Center for Prevention and Control of Important Animal Infectious Diseases and Zoonoses, Yangzhou University, Yangzhou 225009, China

**Keywords:** porcine deltacoronavirus, membrane protein, monoclonal antibody, antigenic epitope

## Abstract

Porcine deltacoronavirus (PDCoV) is an emerging virus that poses a significant threat to the global swine industry. Its membrane (M) protein is crucial for virion assembly and virus–host interactions. We selected the hydrophilic region of M protein for prokaryotic expression, purification, and recombinant protein production. Utilizing hybridoma technology, we prepared the monoclonal antibody (mAb) 24-A6 against M protein. The mAb 24-A6 was shown to be suitable for use in immunofluorescence assays, western blotting, and immunoprecipitation, with specificity for PDCoV and no cross-reactivity with other five porcine viruses. The M protein was observed to be expressed as early as 3 h after PDCoV infection, increasing its expression over the duration of infection. Notably, the antigenic epitope of the M protein identified as ^103^SPESRL^108^ recognized by mAb 24-A6 was found within a conserved structural domain (SWWSFNPETNNL) of the coronavirus M protein, indicating a crucial overlap between a functionally important viral assembly region and a region recognized by the immune system. Our findings provide valuable insights into mAb 24-A6 targeting the antigenic epitope of M protein and may contribute to the development of diagnostic tools for PDCoV infection and fundamental research into the function of PDCoV M protein.

## 1. Introduction

Porcine deltacoronavirus (PDCoV) is a member of the genus *Deltacoronavirus* within the subfamily *Orthocoronavirinae* of the family *Coronaviridae* [1] and was first identified in Hong Kong in 2012 [1]. A large-scale outbreak of unexplained diarrhea in sows and piglets in the United States in 2014 was later confirmed to be caused by PDCoV infection [2]. Subsequently, cases of PDCoV infection were detected in Canada [3], South Korea [4], China [5], Thailand [6], Laos [7], Vietnam [8], Japan [9], Mexico [10], and Peru [11], causing significant economic losses to the swine industry. 

Unlike other swine viruses, PDCoV has the potential for cross-species transmission. There is evidence that PDCoV can cross species barriers and infect non-porcine hosts. PDCoV has been detected in birds [1], calves [12], poultry [13], and mice [14], suggesting that interspecies transmission of PDCoV may have occurred in nature. Notably, PDCoV has been shown to replicate in human intestinal epithelial cells, suggesting that PDCoV is able to cross the human intestinal barrier and infect human cells [15]. A study published in 2021 found that PDCoV was detected in the plasma of three Haitian children with febrile illnesses, two of whom had coughs and abdominal pain and the other had high fever (40 °C) [16]. These studies demonstrated that PDCoV has the ability to cross species barriers and may have the potential to infect humans and other animals. The increasing prevalence and global spread of PDCoV in commercial pig populations pose potential public health risks associated with cross-species transmission to humans.

PDCoV is primarily transmitted by the fecal–oral route, with contaminated feed and fomites being potential sources of infection [17]. As a type of porcine enteropathogenic coronavirus, PDCoV causes gastrointestinal disease in pigs, often manifesting as watery diarrhea and vomiting in replacement gilts, pregnant sows, and piglets [6]. Upon infection, PDCoV primarily targets the jejunum and ileum, specifically the villous epithelial cells, resulting in villous atrophy and malabsorption [17]. 

PDCoV has an enveloped, positive-sense, single-stranded RNA genome of approximately 25.4 kb [1]. The genome organization of PDCoV is similar to other coronaviruses, with a 5′ untranslated region (UTR), open reading frames (ORFs), encoding viral proteins, and a 3′ UTR. The ORFs are in the following order: 5′-ORF1a/1b-S-E-M-NS6-N-NS7(NS7a)-3′ [18,19]. The M protein is the most abundant protein in viral envelopes [20] and plays a critical role in viral assembly and morphogenesis [21]. The M protein of PDCoV has highly conserved amino acid sequences between different strains. In addition, polyclonal antibodies against the M protein of PDCoV show no cross-reactivity with other coronaviruses [22]. The characteristics of the M protein suggest that it is an ideal candidate protein for the detection of antibodies specific for PDCoV infection. 

Monoclonal antibodies (mAbs) are laboratory-made molecules designed to recognize and bind to specific antigens on the surface of pathogens such as viruses. To date, several B cell epitopes of the PDCoV proteins N [23,24,25,26,27,28,29,30], S [31], NS6 [32], and NS7 [19] have been identified that are either useful for diagnostic purposes or have the neutralizing epitopes for vaccine design potential. However, much work is still needed to fully understand the B cell epitopes of the PDCoV protein and to eliminate non-specific cross-reactivity between coronaviruses by developing differential diagnostic methods for PDCoV. In this study, a truncated M protein of PDCoV was expressed in *Escherichia coli* (*E. coli*), and a hybridoma cell line secreting mAbs against PDCoV M protein was obtained by mice immunization. The specificity of the monoclonal antibody was tested by an indirect immunofluorescence assay (IFA), immunoprecipitation (IP), and western blot. The linear epitope of the PDCoV M protein was further identified, and the expression pattern of the M protein was determined. The results of this study will provide new insights into the mAbs of PDCoV.

## 2. Results

### 2.1. Expression and Purification of the Recombinant Protein

**Figure 1:**
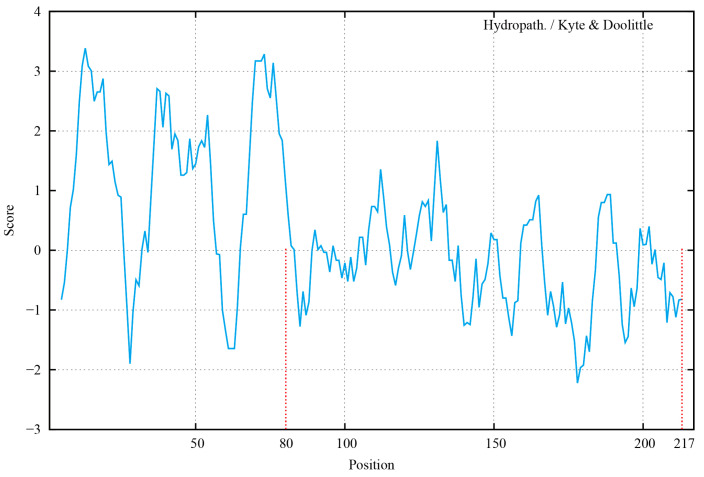
The hydrophilicity plot of the PDCoV M protein. The x-axis represents the amino acid sequence of the M protein; the y-axis represents the degree of hydrophobicity (score > 0) and hydrophilicity (score < 0). The truncated positions of the protein are marked with red dashed lines.

**Figure 2:**
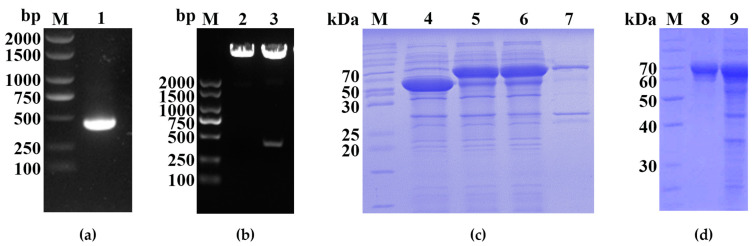
Expression and purification of the recombinant M protein. (**a**) Amplified products of the truncated M gene. Lane M, DL2000 Maker; lane 1, PCR products of the truncated M gene. (**b**) Identification of recombinant plasmids (pCold-TF-M) by enzymatic digestion. M, DL2000 Maker; lane 2, enzymatic cleavage products of pCold-TF; lane 3, enzymatic cleavage products of pCold-TF-M. (**c**) Prokaryotic expression of recombinant M protein. M, protein molecular weight marker (cat# 26614); lane 4, *E. coli* BL21 with empty vector pCold-TF; lanes 5, 6, and 7 represent the whole cell lysate, supernatant, and pellet, respectively, from *E.coli* BL21 transformed with pCold-TF-M (induced with IPTG). (**d**) Purification of the recombinant M protein. M, protein molecular weight marker (cat# 26614); lane 8, the expressed product of pCold-TF-M after purification; lane 9, the whole cell lysate of *E.coli* BL21 transformed with pCold-TF (induced with IPTG).

The results of the hydrophobicity analysis of the PDCoV M protein (Figure 1) showed that the N-terminal region (amino acid positions 80 to 217) of the M protein was highly hydrophilic and was selected for primer design. The truncated M gene fragment (Figure 2a, 417 bp) was amplified using the PDCoV GX2021-1 strain and inserted into the pCold-TF plasmid. The restriction enzyme digestion product was identified by nucleic acid electrophoresis (Figure 2b). The recombinant plasmid pCold-TF-PDCoV-M was transformed into BL21(DE3) competent cells, and the recombinant protein expression was induced with IPTG at 16 °C for 12 h. The cells were lysed, the target protein was extracted from supernatant using SDS-PAGE, and the results showed that there was a clear 60–70 kDa target protein band of the expected size (Figure 2c). The target protein was recovered by gel excision. The recombinant PDCoV-M protein was then purified through a series of processes including the electrophoretic elution of proteins, salt ion equilibration, and concentration (Figure 2d). 

### 2.2. Preparation, Production, and Characterization of mAbs

BALB/c mice were immunized with recombinant M protein, and splenocytes were harvested and fused with SP2/0 cells. A stable hybridoma cell line secreting monoclonal antibodies against PDCoV M protein, named 24-A6, was obtained through a rigorous selection and subcloning process. Two BALB/c mice were pre-injected with 500 μL of liquid paraffin into the peritoneal cavity seven days prior. The cultured 24-A6 cell line (approximately 2.5 × 10^6^ cells) was then injected into the peritoneal cavity of the mice. When abdominal distension was evident (approximately seven days), the abdomen was punctured with a syringe needle, and the ascites were collected in an Eppendorf tube. The samples were then centrifuged at 1500 rpm for 10 min, and the supernatant was collected and stored at −20 °C. Compared to hybridoma supernatant-derived mAb, mouse ascites fluid mAb, used in all experiments except for mAb screening, is less expensive and has higher titers.

**Figure 3:**
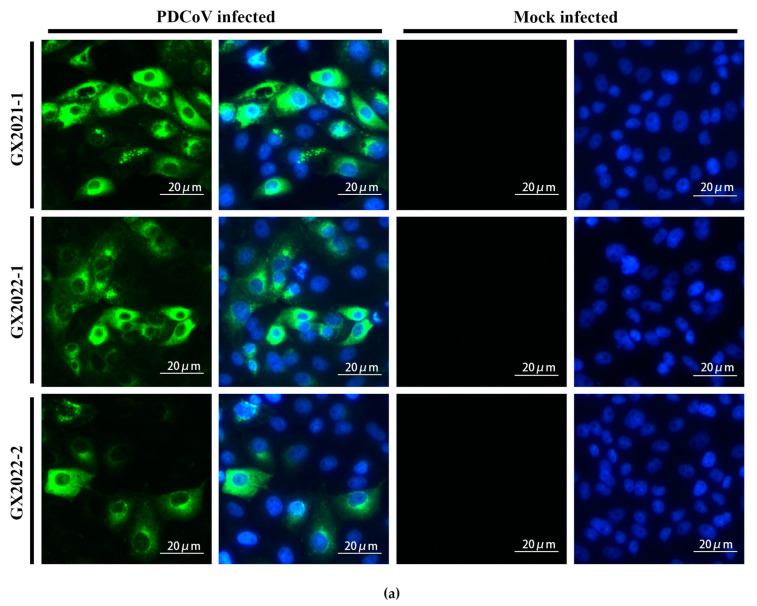

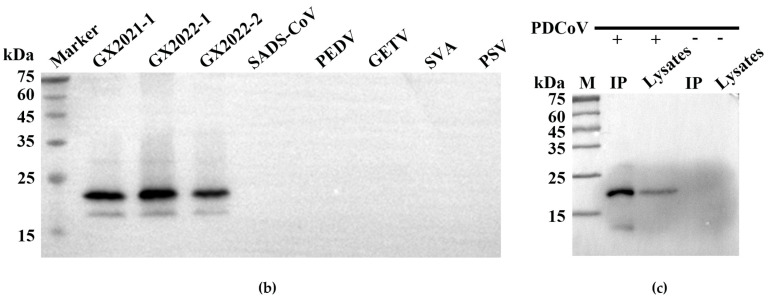
Identification of mAb against PDCoV M protein. (**a**) IFA detection of mAb 24-A6 in PDCoV-infected cells. The green fluorescence represents the reaction of mAb 24-A6 with different strains of PDCoV. The blue fluorescence represents the nucleus of LLC-PK1 cells. (**b**) Western blot detection of monoclonal antibodies. The reactivity of mAb 24-A6 with GX2021-1, GX2022-1, and GX2022-2 strains of PDCoV-, SADS-CoV-, PEDV-, GETV-, SVA-, and PSV-infected cells was analyzed by western blotting. (**c**) Detection of the capacity of mAb 24-A6 for co-immunoprecipitation (Co-IP). Lane M, protein molecular weight marker; lane Lysates, total cell lysate; lane IP, immunoprecipitated fraction.

Three PDCoV strains (GX2021-1, GX2022-1, and GX2022-2) maintained in our laboratory were each used to infect LLC-PK1 cells, and the mouse ascites fluid mAb 24-A6 was used as the primary antibody for detection. The results showed that all three PDCoV-infected cells reacted with mAb 24-A6 (Figure 3a), indicating that mAb 24-A6 can be used to detect PDCoV strains.

Western blot assays were performed using mAb 24-A6 as the primary antibody on cell samples from three PDCoV-infected LLC-PK1 cells and SADS-CoV (swine acute diarrhea syndrome coronavirus)-, PEDV (porcine epidemic diarrhea virus)-, GETV (getah virus)-, SVA (senecavirus A)-, and PSV (porcine sapelovirus)-infected ST cells, respectively. The results showed that a clear target protein band (approximately 24.6 kDa) was detected in the lanes of all three PDCoV-infected LLC-PK1 cells (Figure 3b), whereas no specific bands were detected in the lanes of SADS-CoV-, PEDV-, GETV-, SVA-, and PSV-infected ST cells, indicating good specificity of the monoclonal antibody.

After PDCoV infection of LLC-PK1 cells, the cells were harvested, lysed, and processed into antigen–antibody complexes together with monoclonal antibodies, and then magnetic beads were added to prepare magnetic bead–antigen–antibody complexes for detection by western blotting. The results showed that specific target bands were detected in the lanes of PDCoV-infected cell samples, and no specific target bands were detected in the lanes of cell samples not infected with PDCoV, indicating that the monoclonal antibody could enrich more target antigens (Figure 3c).

### 2.3. M protein Expression in PDCoV-Infected Cells at Different Time Points

**Figure 4:**
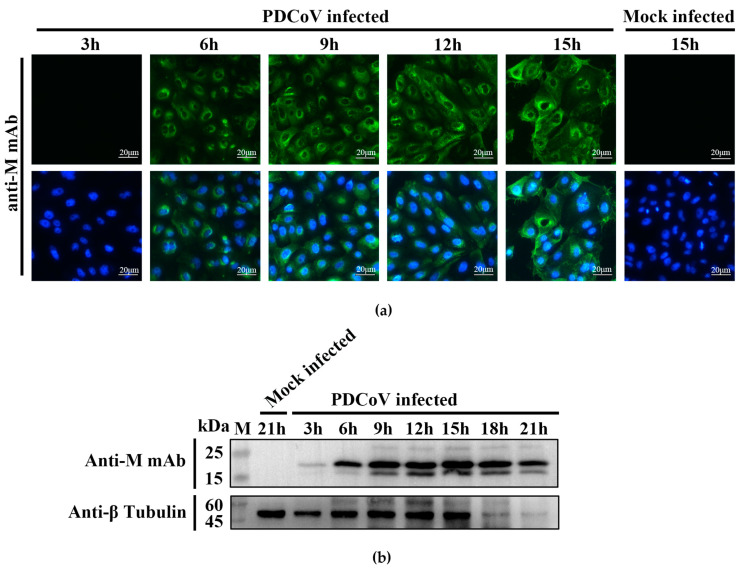
Detection of M protein expression at different time points after PDCoV infection. (**a**) M protein expression of PDCoV at different time points was detected by IFA. (**b**) M protein expression of PDCoV at different time points was detected by WB.

PDCoV was used to infect LLC-PK1 cells, and then immunofluorescence and western blot detection were performed at different time points after infection using mAb 24-A6 as the primary antibody. IFA results showed that PDCoV M protein was detected at 6 h after infection (Figure 4a). As the time of PDCoV infection increased, M protein expression increased, and cell detachment, death, and rounding became more severe. Western blot results showed that M protein was expressed at 3 h after infection, and the expression level of M protein increased with the duration of infection (Figure 4b). The IFA and western blot assays indicated that M protein was expressed at the early stage of PDCoV infection and continued to be expressed until cell death occurred.

### 2.4. Epitope Identification

**Figure 5:**
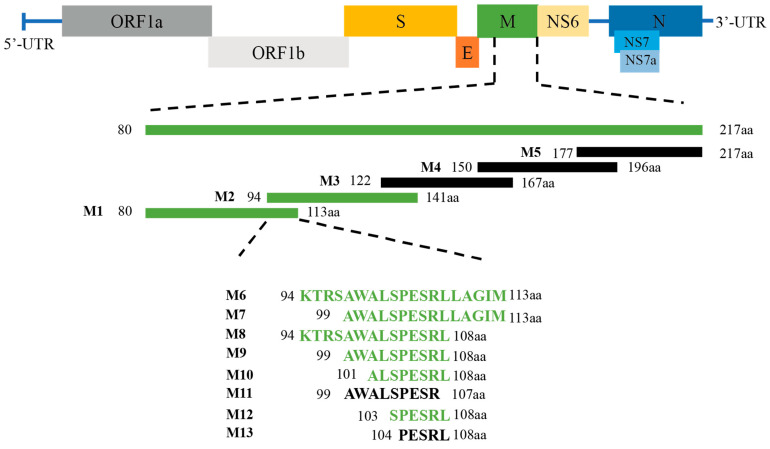
Schematic of the PDCoV M protein truncation used for B cell epitope mapping. The green fragments indicate that the peptides reacted with mAb 24-A6. The black fragments indicate that the peptides did not react with mAb 24-A6.

**Figure 6:**
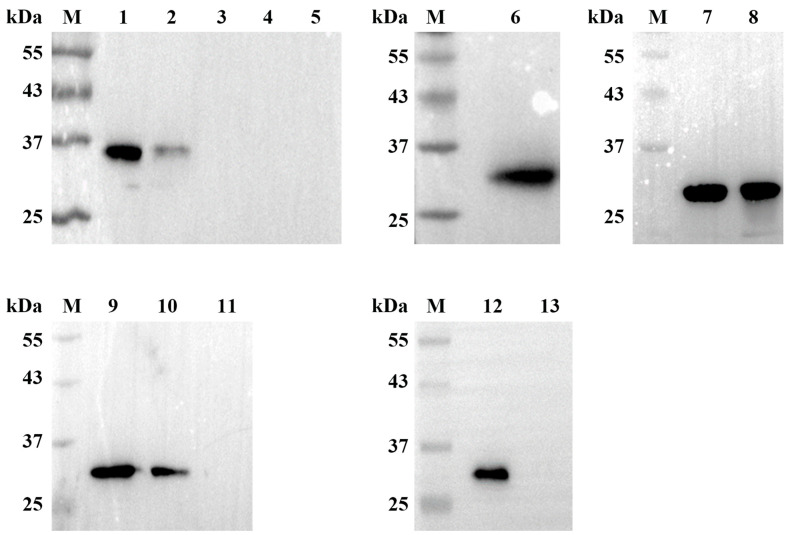
Identification of the B cell epitope of the PDCoV M protein. A series of truncated M fragments were cloned into plasmid pEGFP-C3 and expressed in HEK 293T cells. Thirteen peptides (peptide M1–13) were expressed to detect the minimal epitope and tested for reactivity with mAb 24-A6 by western blotting. Lane M, tricolor pre-dyed protein molecular weight marker; lane 1, peptide 80–113 aa (M1); lane 2, peptide 94–141 aa (M2); lane 3, peptide 122–167 aa (M3); lane 4, peptide 150–196 aa (M4); lane 5, peptide 177–217 aa (M5); lane 6, peptide 94–113 aa (M6); lane 7, peptide 99–113 aa (M7); lane 8, peptide 94–108 aa (M8); lane 9, peptide 99–108 aa (M9); lane 10, peptide 101–108 aa (M10); lane 11, peptide 99–107 aa (M11); lane 12, peptide 103–108 aa (M12); lane 13, peptide 104–108 aa (M13).

In order to precisely determine the epitope of the PDCoV M protein recognized by the mAb, the amino acid sequence of the truncated M protein (80–217 aa) was divided into a series of overlapping fragments (Figure 5). The protein truncation assay showed that peptides M1 and M2 were recognized by mAb 24-A6 (presence of specific band in western blot) (Figure 6), confirming that the epitope was in the overlapping sequence (peptide M6). Further truncation of peptide M6 from both termini finally revealed that the loss of a single amino acid from either the C-terminus of peptide M11 or the N-terminus of peptide M13 prevented recognition by mAb 24-A6. Consequently, peptide ^103^SPESRL^108^, localized to peptide M12, was the minimal linear epitope for binding mAb 24-A6 (Figure 6).

### 2.5. Spatial Localization and Conservation of Epitope

**Figure 7:**
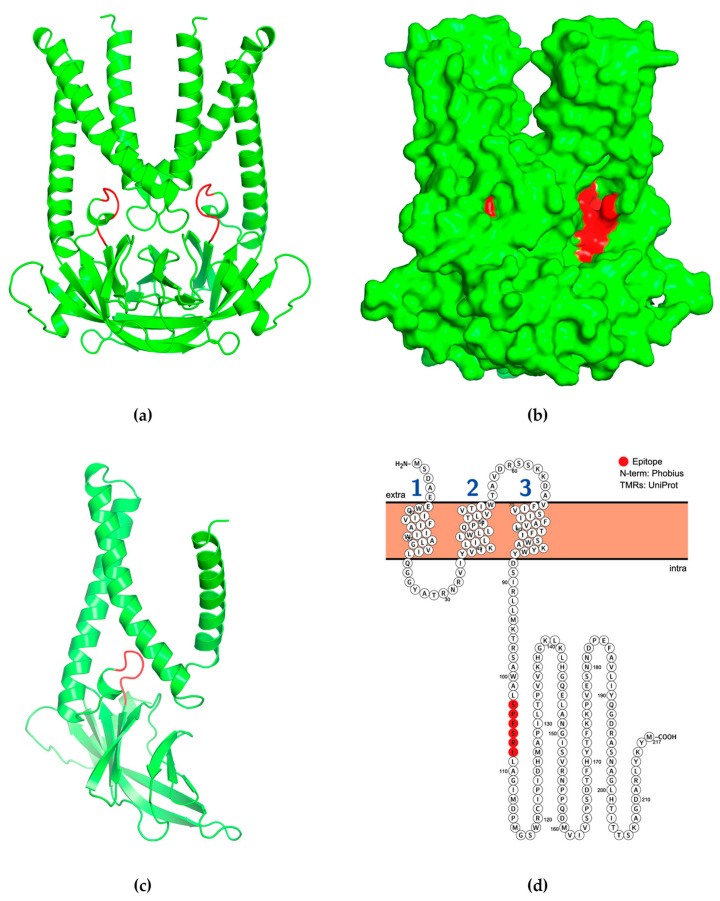
The predicted 3D structure and transmembrane topology of M protein. The position of the antigenic epitope ^103^SPESRL^108^ is marked in red. (**a**) The predicted cartoon model structure of M symmetric homodimer. (**b**) The predicted surface model structure of M symmetric homodimer. (**c**) The predicted cartoon model structure of M protein monomer. The PDCoV M protein has a triple helix bundle. (**d**) The predicted transmembrane topology of PDCoV M protein. The PDCoV M protein forms three transmembrane domains. The antigenic epitope ^103^SPESRL^108^ of PDCoV M protein is located in the intracellular region.

The 3D structural models of the PDCoV M protein were predicted using AlphaFold2 and rendered using PyMOL. Based on our prediction models, the antigenic epitope ^103^SPESRL^108^ was located below the transmembrane helix of the M protein (Figure 7a–c), specifically on the intracellular side of the membrane (Figure 7d). 

**Figure 8:**
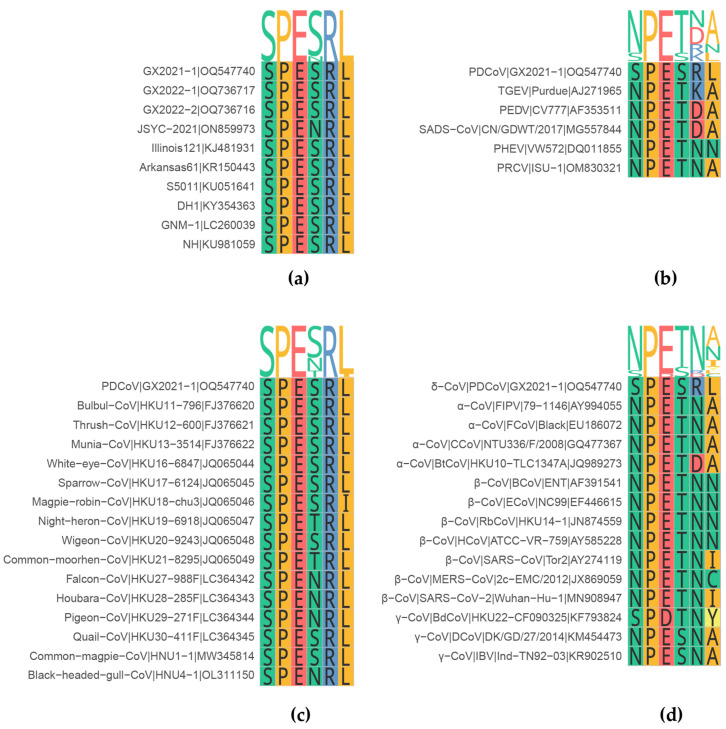
Comparison of the epitope sequences recognized by mAb 24-A6 among different coronaviruses. Alignment of the epitope sequences recognized by mAb 24-A6 with PDCoV reference strains (**a**), porcine coronavirus reference strains (**b**), deltacoronavirus reference strains (**c**), and coronavirus reference strains (**d**).

The M protein sequences of all 240 PDCoV strains available in July 2023 were downloaded from GenBank and aligned. The alignment result showed that the amino acid sequence of the ^103^SPESRL^108^ antigenic epitope region was identical in all PDCoV strains, except for the Ser106Asn mutation of strain JSYC-2021 (Genbank ID: ON859973) (Figure 8a and Figure S1). This finding suggests that the ^103^SPESRL^108^ sequence is a conserved epitope on the PDCoV M protein. The alignment results of six porcine coronaviruses showed that PDCoV and the other five porcine coronaviruses shared 33.3% sequence similarity with epitope recognized by mAb 24-A6 (Figure 8b). In addition, sequence alignment of the epitope recognized by mAb 24-A6 with 15 deltacoronaviruses was performed to determine the degree of conservation among deltacoronaviruses. The results showed 83%~100% sequence similarity with the epitope recognized by mAb 24-A6 (Figure 8c). In addition, sequence analysis of 14 different coronaviruses, including α, β, γ, and δ coronaviruses, showed that the epitope recognized by the mAb 24-A6 shared 33% similarity with other coronaviruses and that only two residues ^104^PE^105^ were relatively conserved among all the selected coronaviruses (Figure 8d).

### 2.6. Analysis of mAb 24-A6 Light and Heavy Chain Sequences

**Figure 9:**
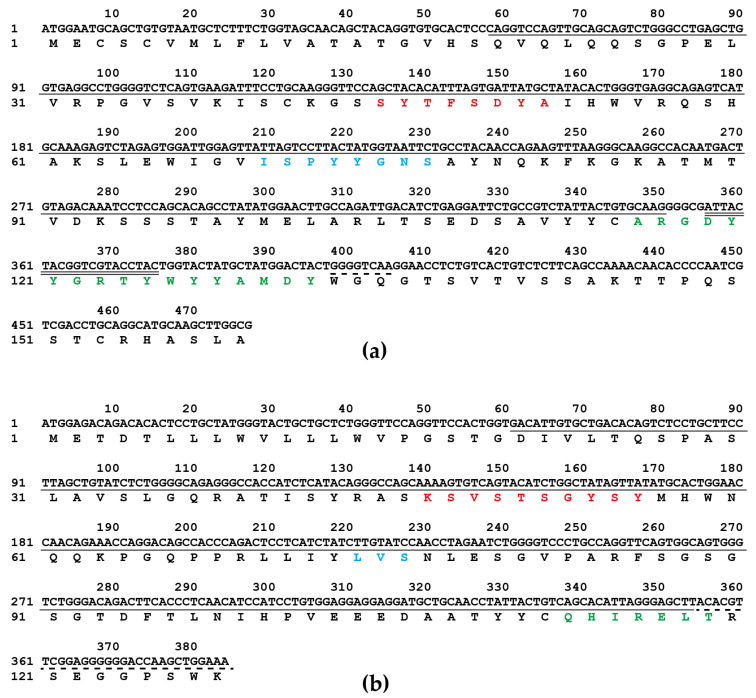
The nucleotide sequences and the corresponding amino acid sequences of the VH (**a**) and VL (**b**) chain regions. The complementarity-determining region 1 (CDR1), CDR2, and CDR3 are highlighted in red, blue, and green, respectively. Solid, double, and dotted lines represent V, D, and J regions, respectively.

Agarose gel electrophoresis showed a single primary band of the expected size for both the variable region of heavy (VH) and light (VL) chains of mAb 24-A6 (Figure S2). Sequencing results revealed that the VH gene of mAb 24-A6 spans 477bp, encoding 159 amino acids, while the VL gene spans 384bp, encoding 128 amino acids (Figure 9). IGBLAST analysis indicated that the light and heavy chain genes of mAb 24-A6 have a high degree of similarity (98.3% and 94.5%, respectively) to murine antibody sequences, with their gene sequences fitting the variable region framework structure of murine Ig. The V, D, and J segments of the mAb 24-A6 VH gene aligned most closely with the IMGT database reference sequences IGHV1-67*01 and IGHV1S137*01, IGHD1-1*01, and IGHJ4*01, respectively. Similarly, the V and J segments of the mAb 24-A6 VL gene showed the highest alignment to the IMGT database reference sequences IGKV3-12*01 and IGKJ2*01, respectively. These results suggest that the VH and VL genes of mAb 24-A6 are likely to have been generated from these germline gene segments by V-(D)-J recombination.

## 3. Discussion

Since the emergence of PDCoV in late 2012, the global swine industry has been grappling with the challenges posed by the PDCoV pandemic. A critical aspect of PDCoV pandemic management has been the development of diagnostic tools to contain the spread of the virus. However, the clinical signs of pigs infected with PDCoV are very similar to those caused by infection with PEDV and TGEV, and mixed infections are common in swine herds [33]. This adds to the difficulty in making a preliminary assessment of this pathogen. In addition, although PDCoV primarily infects pigs, its genetic proximity to other coronaviruses (e.g., SARS-CoV-2) gives it the ability to infect other animals, such as birds [1], cattle [12], mice [14], and humans [16], necessitating continued surveillance of PDCoV to prevent potential zoonotic spillover.

Several mAbs have been prepared against PDCoV proteins, primarily targeting the PDCoV nucleocapsid (N) protein [23,24,25,26,27,28,29,30]. However, the rapid mutation rate of the virus can lead to antigenic drift, allowing it to evade specific mAbs, thereby reducing the detection efficiency of these mAbs. The M protein, which plays a critical role in viral assembly [21], virus host–cell interactions [34], budding [35], and immune evasion [36], could serve as another potential target for mAbs. M protein-based immunodiagnostics may provide accurate and effective detection methods for PDCoV infection. By combining multiple mAbs targeting different epitopes of both N and M viral proteins, mAb cross-reactivity and viral immune escape could be minimized. Therefore, further investigation of mAbs against PDCoV M protein is warranted.

The western blot results showed a distinct band at approximately 24.6 kDa, corresponding to the size of the PDCoV M protein, in the lanes from all three PDCoV-infected LLC-PK1 cells (Figure 4b). The absence of specific bands in the lanes from SADS-CoV-, PEDV-, GETV-, SVA-, and PSV-infected cells suggests that mAb 24-A6 specifically recognizes the PDCoV M protein, demonstrating its specificity (Figure 4b). Meanwhile, the sequence alignment results confirmed the western blot results. The M protein sequences of 240 PDCoV strains showed that the ^103^SPESRL^108^ antigenic epitope, which was the target of mAb 24-A6, was conserved in all but one strain (Figure 8a and Figure S1). Whether the minor variant (Ser106Asn mutation) of M proteins in strain JSYC-2021 (GenBank ID: ON859973) can be recognized by mAb 24-A6 requires further investigation. The conservation of this epitope in the majority of PDCoV strains indicates a high degree of specificity of mAb 24-A6 for PDCoV. Further analysis revealed 33.3% sequence similarity between the mAb 24-A6 epitope and the corresponding region in five porcine coronaviruses (Figure 8b), and 83–100% similarity among fifteen deltacoronaviruses (Figure 8c). These results suggest that mAb 24-A6 may also recognize some deltacoronaviruses but is unlikely to cross-react with other porcine coronaviruses, as confirmed by the western blot experiments mentioned above. The 33% similarity to other coronaviruses and the conservation of only two residues (^104^PE^105^) among all the selected coronaviruses further confirms the specificity of mAb 24-A6 for PDCoV (Figure 8d). In brief, the western blot and sequence alignment results support each other, demonstrating the specificity of mAb 24-A6 for PDCoV among six viruses tested. This specificity is due to the high conservation of the ^103^SPESRL^108^ antigenic epitope recognized by mAb 24-A6. The reactivity of this monoclonal antibody 24-A6 with other viruses remains to be investigated.

It should be noted that the ^103^SPESRL^108^ epitope in the PDCoV M protein is located within the conserved SWWSFNPETNNL domain of coronaviruses. The conserved SWWSFNPETNNL domain is important for virus assembly, and its conservation across coronaviruses suggests a universal role in the virus life cycle [37]. Within this domain, the ^103^SPESRL^108^ epitope of PDCoV is recognized by mAb 24-A6. The conservation of this epitope across PDCoV strains suggests its importance for immune recognition. The overlap of these two regions suggests that the antibody recognition site (^103^SPESRL^108^) is located in a critical region responsible for virus assembly. This could have important implications for both diagnostics and therapeutics. For diagnostics, the conserved SWWSFNPETNNL domain containing the ^103^SPESRL^108^ epitope could be used for preparing mAbs due to the conservation of this region. For therapeutics, antibodies targeting this region could disrupt viral assembly, thereby inhibiting viral replication and spread. Further studies are needed to confirm these hypotheses. In particular, experimental work could determine whether mAb 24-A6 cross-reacts with other coronaviruses and whether antibodies targeting ^103^SPESRL^108^ can inhibit virus assembly.

In summary, a mAb 24-A6 against PDCoV M protein was prepared, which showed specificity for PDCoV and suitability for various diagnostic applications. The epitope ^103^SPESRL^108^ recognized by mAb 24-A6 was highly conserved among PDCoV strains and was found to be located within a conserved structural domain of coronavirus, suggesting a crucial relationship between a viral assembly region and an immune-recognized region. This study provides a fundamental tool for the establishment of new PDCoV diagnostic methods and further research into the function of the PDCoV M protein.

## 4. Materials and Methods

### 4.1. Viruses, Cells, and Animals

Three PDCoV strains (GX2021-1, GX2022-1, and GX2022-2) were isolated and maintained in our laboratory. Human embryonic kidney (HEK293T) cells, murine myeloma (SP2/0) cells, and porcine kidney (LLC-PK1) cells were cultured in Dulbecco’s modified Eagle’s medium (DMEM) (Gibco, Waltham, MA, USA) supplemented with 10% fetal bovine serum (Sigma, St. Louis, MO, USA) at 37 °C with 5% CO_2_. Six-to-eight-week-old BALB/c mice were obtained from the Animal Experimental Center of Yangzhou University.

### 4.2. Expression and Purification of the PDCoV M Protein

The amino acid sequence (GenBank ID: WFD49995) of the PDCoV M protein was obtained from GenBank, and the hydrophilic regions of the PDCoV M protein were analyzed using ProtScale (https://web.expasy.org/protscale/, accessed on 1 May 2021). Primers were designed to amplify the nucleotide sequence of the hydrophilic region of the M protein at positions 80 to 217. The primer sequences used in this study are listed in Table 1. The recombinant plasmid pCold-TF-PDCoV-M (TF-M) was constructed by ligating the truncated PDCoV M gene into the vector at the EcoR I and Hind III sites, and the plasmid was subsequently transformed into BL21 (DE3) competent cells. The recombinant M protein was induced with 0.2 mM isopropyl-β-D-thiogalactopyranoside (IPTG) for 12 h at 16 °C, and the fusion protein was then separated by sodium dodecyl sulfate–polyacrylamide gel electrophoresis (SDS-PAGE). The recombinant M protein was stained with 0.25 M KCl and purified by gel excision.

### 4.3. Generation and Screening of mAbs against PDCoV M Protein

Purified recombinant M protein and Freund’s adjuvant (Sigma, St. Louis, MO, USA) were mixed in equal volumes and then injected into 6–8-week-old BALB/c mice. Mice were immunized at two-week intervals, and serum antibodies were detected by immunofluorescence on day 9 after the third immunization. Mice with strong immunofluorescence were used for the final booster immunization and cell fusion. Mouse splenocytes were harvested on day 3 after the final booster immunization and mixed with pre-prepared myeloma cells in a 4:1 ratio for cell fusion in the presence of PEG1500 (Sigma, St. Louis, MO, USA). Positive cell clones were screened by IFA, and two rounds of subcloning were performed using the limited dilution method. To obtain monoclonal cells, the antibody-secreting hybridoma cells were injected intraperitoneally into mice sensitized to liquid paraffin, and the ascites of mice were collected after 7 days.

### 4.4. Indirect Immunofluorescence Assay

LLC-PK1 cells were seeded in a 96-well plate and grown to 80–90% infected with PDCoV-GX2021-1 strain. The original culture medium was discarded, and the cells were washed twice with PBS. PDCoVs diluted in a medium containing trypsin were added to the cells and incubated at 37 °C with 5% CO_2_ for 12 to 24 h. The medium was removed when approximately 50% of the cells were cytopathic. The cells were permeabilized with a 0.1% Triton X-100 solution for 20 min, fixed with a pre-cooled 4% paraformaldehyde solution for 15 min, and then blocked with a 2% BSA solution for a further 30 min. Hybridoma cell culture supernatants were used as primary antibodies and added to each well. Sera from unvaccinated mice were used as a negative control and PDCoV-N monoclonal antibody stored in our laboratory was used as a positive control, and both were incubated overnight at 4 °C. A 1:1000 dilution of 488 labeled goat anti-mouse IgG was added to each well and incubated for 1 h at room temperature. For each of the above steps, each well was washed twice with PBS for 2 min. The results were observed under an inverted fluorescence microscope and photographed for storage.

### 4.5. Western Blot Identification of mAbs

LLC-PK1 cells were seeded in 12-well plates and grown to 80–90% confluence before inoculation with PDCoV. After 12–24 h of infection, cells were harvested, lysed, and mixed with a loading buffer, heated in a metal bath at 100 °C for 10 min, and then subjected to SDS-PAGE for isolation. The PVDF membrane was activated by soaking in methanol for 2 min, and the gel was transferred to the PVDF membrane at 200 mA for 40 min. The membrane was blocked with a 5% non-fat milk TBST solution for 2 h at room temperature. The mAb against the M protein prepared in this study was used as the primary antibody at a dilution of 1:1000 and incubated overnight at 4 °C on a rotary platform. HRP-conjugated goat anti-mouse IgG was used as the secondary antibody at a dilution of 1:10,000 and was incubated for 1 h at room temperature on a rotary platform. For each of the above steps, the membrane was washed twice with TBST for 10 min at room temperature. The ECL reagent was added, and the membrane was exposed to an imager for detection.

### 4.6. Identification of mAbs by Immunoprecipitation

LLC-PK1 cells were seeded in 3.5 cm cell culture dishes and grown to 80% confluence before inoculation with PDCoV. Cells were harvested and lysed after 12–16 h and 30% cell cytopathy. Magnetic bead–antibody–antigen complexes were prepared, according to the IP experiment protocol, and 100 µL of a 1× loading buffer was added. Samples were boiled at 100 °C for 10 min and subjected to western blot detection.

### 4.7. Investigating M Protein Expression Using mAb

To investigate M protein expression during PDCoV infection, LLC-PK1 cells were inoculated at a multiplicity of infection (MOI) of 0.1, and cell samples were collected at 3, 6, 9, 12, 15, 18, and 21 h post-infection. M protein expression was detected by IFA and WB using mAb 24-A6.

### 4.8. Precise Epitope Mapping of the PDCoV M Protein

To determine the precise epitope of the PDCoV M protein recognized by the mAb, the amino acid sequence of the truncated M protein (80–217 aa) was divided into five overlapping fragments (Figure 5) and cloned into the pEGFP-C3 vector (restriction enzyme sites: Hind III and EcoR I). The primers used for the truncated fragments are listed in Table 1. In addition, the overlapping region (94–113 aa, designated peptide M6) of peptide M1 (80–113 aa) and peptide M2 (94–141 aa) was cloned into the pEGFP-3C plasmid. The amino acid sequence of fragment M6 was then further reduced from both the N- and C-termini. For N-terminal reduction, the upstream primer of the fragment (introducing restriction enzyme site: Hind III) and the downstream primer of pEGFP-C3 (restriction enzyme site: Mlu I) were designed. For C-terminal reduction, the upstream primer of pEGFP-C3 (restriction enzyme site: Nhe I) and the downstream primer of the fragment (introducing restriction enzyme site: BamH I) were designed. The recombinant plasmids were transfected into HEK293T cells, and the expression of the truncated protein was observed by fluorescence microscopy, followed by western blotting to detect the reactivity of the truncated proteins with mAb.

### 4.9. M Protein Structure and Epitope Distribution

The theoretical three-dimensional structure of the M protein homodimer of PDCoV (strain CHN-GD16-05, accession number KY363868) was predicted using AlphaFold2 software (version 2.3.1) [38]. The amino acid sequence of the M protein was submitted to the ColabFold program (version 1.5.2) [39], which used MMseqs2 for rapid homology search and invoked AlphaFold2 for structure prediction. The predicted three-dimensional structure of the M protein was displayed using PyMOL (open-source version 2.5.0) [40]. To determine the putative transmembrane profile, a residue-based diagram of the M protein was created. Information on the transmembrane domains in the M protein was obtained from the UniProt database (https://www.uniprot.org/, accessed on 1 August 2023). Prediction of the transmembrane topology was performed using Phobius (version 1.01) [41]. The organization of the transmembrane domains in the M protein was visualized using Protter (version 1.0) [42]. 

### 4.10. Epitope Sequence Similarity Analysis

Sequence conservation of the epitope recognized by mAb 24-A6 was analyzed at the level of PDCoV, porcine coronavirus, deltacoronavirus, and coronavirus. The genome sequences of 240 PDCoVs, 5 porcine coronaviruses, 15 deltacoronaviruses, and 14 coronaviruses were downloaded from GenBank. The M protein epitope and flanking sequences were compared with other selected coronavirus strains using MACSE (version 2.03), and the ggmsa package (version 1.4.0) [43] of R software (version 4.2.3) [44] was used to visualize the multiple sequence alignment and sequence logo. 

### 4.11. Cloning of the Variable Regions of the mAb 24-A6 Heavy and Light Chains 

Total RNA was extracted from hybridoma cell lines secreting mAb 24-A6 using an RNA extraction kit (TIANGEN, Beijing, China), and subsequently reverse transcribed into cDNA using a reverse transcription kit (Vazyme, Nanjing, China). The VH and VL chains of mAb 24-A6 were amplified using the primers listed in Table 1. Following purification and recovery via agarose gel electrophoresis, the resulting heavy and light chain fragments were ligated into the pMD19-T vector, respectively. Positive clones were subsequently identified, and their plasmids were sequenced.

## Figures and Tables

**Table 1:** Primers used in this study.

Primer Names	Primer Sequence ^1^ (5′–3′)		Size (bp)
	Sense	Negative Sense	
M protein	ATCCGAATTCATATCCTGGGCCAAGTAC	TCGACAAGCTTTTACATATACTTATACAGGC	414
M1	TCAAGCTTATATCCTGGGCCAAGTACTGG	GAATTCCATAATCCCTGCAAGGAGTCTACTCTC	102
M2	CAAGCTTATGAAAACCAGATCTGCATGGGC	GAATTCCTTGAGCTTGCCATGCTTAACGACTG	144
M3	AAGCTTATTCCCATCGACCACATGGCTCCA	GAATTCGTCACTTGGTGACACTATCACCATATCC	138
M4	AAGCTTGCCAATGGCATATCAGTCAGAAAT	GAATTCTGAAGCGCGGTCACCCTGGTATAT	141
M5	GAATTCTGAAGCGCGGTCACCCTGGTATAT	CAGAATTCTTACATATACTTATACAGGCGAGCG	123
M6	CAAGCTTATGAAAACCAGATCTGCATGGGC	GAATTCCATAATCCCTGCAAGGAGTCTACTCTC	60
M7	CCCAAGCTTTCTGCATGGGCACTCTCACCTGAG	GAATTCCATAATCCCTGCAAGGAGTCTACTCTC	45
M8	CGGGATCCGAGTCTACTCTCAGGTGAGAGT	CGGGATCCGAGTCTACTCTCAGGTGAGAGT	45
M9	CCCAAGCTTTCTGCATGGGCACTCTCACCTGAG	CGGGATCCGAGTCTACTCTCAGGTGAGAGT	30
M10	CCCAAGCTTTGGGCACTCTCACCTGAGAGTAGA	CGGGATCCGAGTCTACTCTCAGGTGAGAGT	24
M11	CCCAAGCTTTCTGCATGGGCACTCTCACCTGAG	CGGGATCCTCTACTCTCAGGTGAGAGTGCCCA	27
M12	CCCAAGCTTTCACCTGAGAGTAGACTCGGATCCAC	CGGGATCCGAGTCTACTCTCAGGTGAGAGT	18
M13	CCCAAGCTTCCTGAGAGTAGACTCGGATCCACC	CGGGATCCGAGTCTACTCTCAGGTGAGAGT	15
pEGFP	ATCCGCTAGCGCTACCGGTCGCCA	CAATTTACGCGTTAAGATACATTGATG	
VL	ATGGAGACAGACACACTCCTGCTAT	GGATACAGTTGGTGCAGCATCAGCCCGTTT	384
VH	ATGGRATGSAGCTGKGTMATSCTCT	TGGGGSTGTYGTTTTGGCTGMRGAGACRGTGA	477

^1^ Restriction sites are shown as underlined.

## Data Availability

The data supporting the findings of this study are available within the article.

## Supplementary Materials

The supporting information can be downloaded at https://www.mdpi.com/article/10.3390/ijms241813934/s1.
