# Supplementary material for: Identification of a Monoclonal Antibody against Porcine Deltacoronavirus Membrane Protein
Source: Int J Mol Sci. 2023 Sep 11;24(18):13934. doi: 10.3390/ijms241813934 (PMC10530725; doi:10.3390/ijms241813934)
Supplement: Supplementary file 1 [file ijms-24-13934-s001.zip › ijms-2579144-supplementary.pdf]

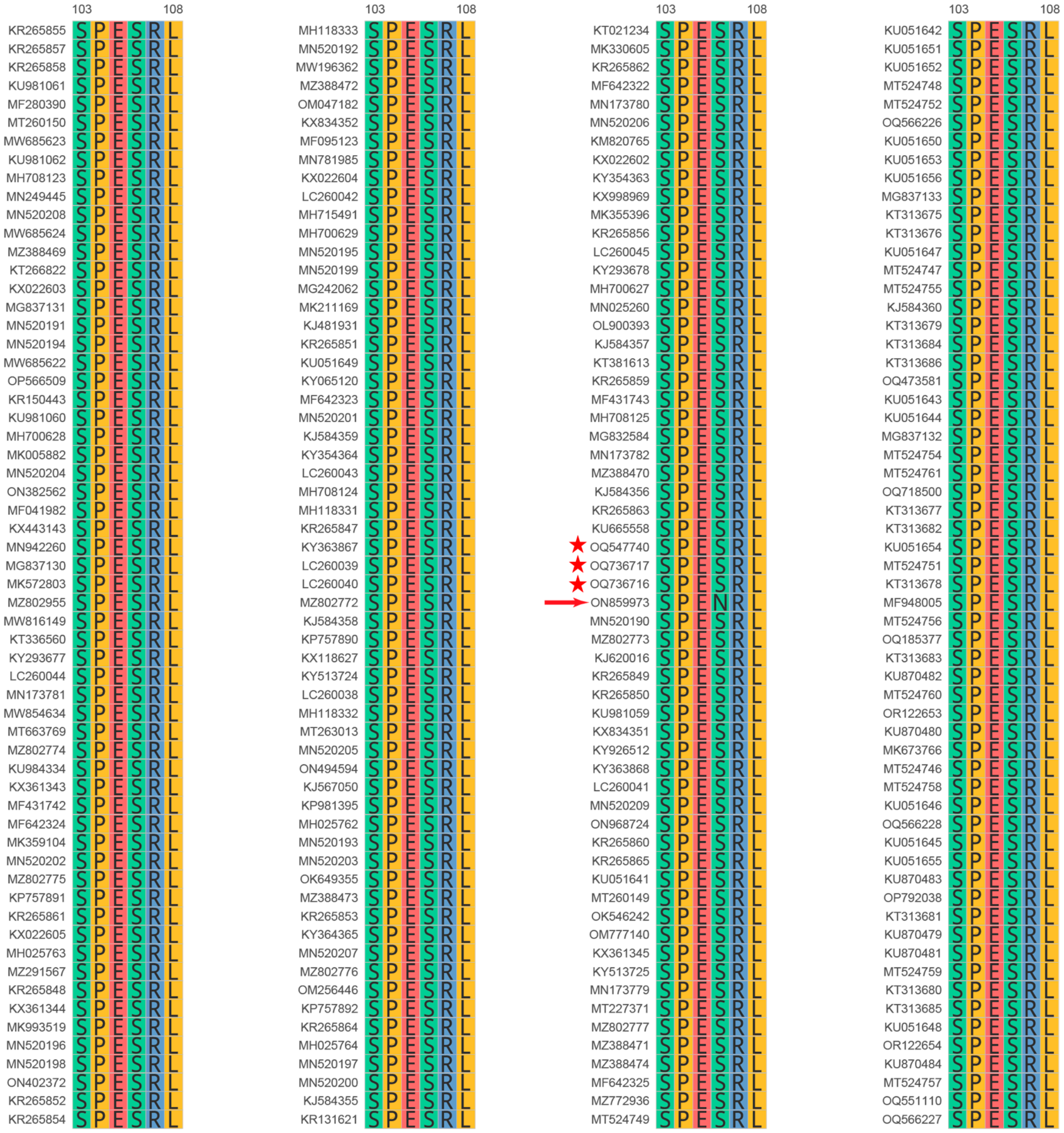

Figure S1. Comparison of the epitope sequences recognized by mAb 24-A6 among 240 PDCoV strains. Red pentagrams indicate the strains used in this experiment. Red arrow indicates the strain with mutation.

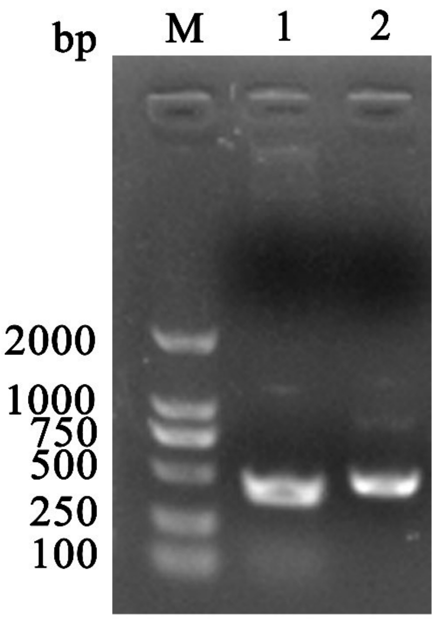

Figure S2. Amplified products of the VH and VL genes of mAb 24-A6. Amplified products of the truncated M gene. Lane M, DL2000 Maker; lane 1, PCR products of the VL gene; lane 2, PCR products of the VH gene.
